# Autophagic Network Analysis of the Dual Effect of Sevoflurane on Neurons Associated with GABARAPL1 and 2

**DOI:** 10.1155/2020/1587214

**Published:** 2020-06-26

**Authors:** Guolin Lu, Dongdong Rao, Min Zhou, Liangpu Xu, Longxin Zhang, Sujing Zhang, Yuping Wang

**Affiliations:** ^1^Department of Anesthesiology, Fujian Maternity and Child Health Hospital, Affiliated Hospital of Fujian Medical University, 18 Daoshan Road, Fuzhou, 350001 Fujian Province, China; ^2^Department of Clinical Laboratory, Fujian Maternity and Child Health Hospital, Affiliated Hospital of Fujian Medical University, 18 Daoshan Road, Fuzhou, 350001 Fujian Province, China

## Abstract

**Background:**

Sevoflurane is commonly used as a general anesthetic in neonates to aged patients. Preconditioning or postconditioning with sevoflurane protects neurons from excitotoxic injury. Conversely, sevoflurane exposure induces neurotoxicity during early or late life. However, little is known about the underlying mechanism of the dual effect of sevoflurane on neurons. Autophagy is believed to control neuronal homeostasis. We hypothesized that autophagy determined the dual effect of sevoflurane on neurons.

**Methods:**

DTome was used to identify the direct protein target (DPT) of sevoflurane. The STRING database was employed to investigate the proteins associated with the DPTs. Protein-protein interaction was assessed using Cytoscape. WebGestalt was used to analyze gene set enrichment. The linkage between candidate genes and autophagy was identified using GeneCards.

**Results:**

This study found that 23 essential DPTs of sevoflurane interacted with 77 proteins from the STRING database. GABARAPL1 and 2, both of which are DPT- and autophagy-associated proteins, were significantly expressed in the brain and enriched in GABAergic synapses.

**Conclusions:**

Taken together, our findings showed that the network of sevoflurane-DPT-GABARAPL1 and 2 is related to the dual effect of sevoflurane on neurons.

## 1. Background

Sevoflurane is commonly used as a sedative and general anesthesia in neonates to elderly patients. A growing number of preclinical studies have demonstrated that sevoflurane exposure during early life leads to neurodegeneration in rodents and nonhuman primates [1]. Moreover, accumulating evidence has shown that sevoflurane exposure is associated with postoperative cognitive dysfunction in aged patients [2, 3]. Conversely, either preconditioning or postconditioning with sevoflurane has been shown to exert a neuroprotective effect on cerebral ischemia injury in adult rodents [4–6]. Thus, sevoflurane has a dual effect on neurons in the brain in different states. However, the mechanism underlying the dual effect of sevoflurane on neurons remains unclear.

Autophagy plays a crucial role in neuron survival and neurodegenerative diseases, maintaining homeostasis within cells [7, 8]. In response to excitotoxicity, which is characterized by an abrupt and prolonged increase in cytoplasmic Ca^2+^ levels and neurotoxicity [9], which is caused by neurotoxins, emerging evidence strongly suggests that autophagy is triggered to reduce the detrimental effects of excitotoxicity and neurotoxicity [10]. Autophagy induction contributed to protecting against sevoflurane-induced neurotoxicity in elderly patients [11]. Conversely, autophagy blockade ameliorated the cognitive impairment induced by sevoflurane in young mice [12]. In a rat model of focal cerebral ischemia, sevoflurane preconditioning plays a neuroprotective role in reducing Akt signaling activity to activate autophagy [4, 13]. Interestingly, after transient global cerebral ischemia in rats, sevoflurane postconditioning reduces cell apoptosis via activating the JAK-STAT pathway [14]. Recent observations have suggested that STAT3 plays a role in regulating the autophagic process by functioning as an enhancer or suppressor in the nucleus, cytoplasm, and mitochondria [15].

In summary, autophagic activation promotes neurotoxicity in neonatal animals, opposite to exerting a protective effect on neurotoxicity in aged animals. Autophagy induced by sevoflurane preconditioning or postconditioning contributes to protecting against brain injury caused by ischemia and reperfusion. However, no previous study has revealed how autophagy determines the dual effect of sevoflurane on neurons in the brain under different conditions. In recent decades, an increasing number of interesting gene hits have been found using microarray and proteomics and other high-throughput screening assays [16]. Data obtained from humans have contributed to building a network linking a drug and its targets to interacting proteins. DTome, a web-based tool, is used to construct a drug-target interactome [17]. We hypothesized that the dual effect of sevoflurane on neurons in the brain is determined by the autophagic network. In this study, we used DTome to investigate the direct protein targets (DPTs) of sevoflurane and we utilized the STRING database to query protein-protein interactions (PPIs). Furthermore, bioinformatics analysis was used to identify the properties of genes related to the dual effect of sevoflurane.

## 2. Materials and Methods

### 2.1. Sevoflurane Target Query

DTome is composed of such three open-source datasets, namely, DrugBank, PharmGSK, and PINA [17]. Sevoflurane was searched in DrugBank (website: https://www.drugbank.ca/) to find information about sevoflurane and identify its DPTs.

STRING version 10.5 (website: https://string-db.org/cgi/input.pl) was used to generate a PPI network. In the STRING database, the DPTs identified from DTome were searched using a single protein name. The organism was searched as *Homo sapiens*. In the basic settings of the STRINGdb browser, the meaning of network edges was set as evidence (line color indicates the type of interaction evidence). Active interaction sources were set as text mining, experiments, databases, coexpression, neighborhood, gene fusion, and cooccurrence. The minimum required interaction score was set as the medium confidence (0.400), and the score was set as the highest confidence (0.900) for searching gamma-aminobutyric acid receptor-associated protein-like (GABARAPL)1 and 2. The maximum number of interactors was set as 50 in the 1st shell, and none was selected in the 2nd shell. Data regarding the proteins that interacted with the DPTs were exported as simple tabular text from the STRING database. The overall proteins that interacted with the DPTs were verified by deleting duplicated proteins, and the remaining proteins were used to compile a dataset.

### 2.2. Gene Set Enrichment Analysis

WebGestalt 2019 (website: http://www.webgestalt.org/) [18], a web-based gene set analysis toolkit, was used to analyze the enrichment of sevoflurane-associated genes. In basic parameters, the organism of interest was selected as “hsapiens,” which was evaluated using KEGG pathway enrichment analysis. In the gene list, the gene ID type was selected as a gene symbol. The DPTs and DPT-associated genes were uploaded as a gene list. The reference used for enrichment analysis was set as the genome, which was used to determine the Gene Ontology and KEGG enrichment.

### 2.3. Network Visualization

Cytoscape version 3.8.0 (website: http://www.cytoscape.org/), an open-source software platform, was used to visualize the sevoflurane-mediated network. In a text file, sevoflurane-mediated DPTs were compiled as node 1, and DPT-associated genes were compiled as node 2. After the text file was input into Cytoscape, node 1 was set as the source node, and node 2 was set as the target node. DPT-associated genes enriched in the top 10 pathways were selected for investigating the first neighbor outgoing nodes. The significantly targeted genes were further identified as candidate genes.

### 2.4. Identification of Autophagy-Associated Genes

The Human Gene Database (website: http://www.genecards.org/), GeneCards, was used to search for the genomic, transcriptomic, proteomic, genetic, clinical, and functional information of the candidate genes from approximately 125 web sources. The symbol of the candidate gene was searched, and GeneCards provided a great deal of information on the candidate genes, including aliases, expression information, functional genomics, location, pathways, transcripts, domains, and proteins. Based on the functions and pathways of the genes, more information was gathered on the candidate genes associated with autophagy, including their genomics, location, domains, proteins, and expression. Kyoto Encyclopedia of Genes and Genomes (KEGG) database was used to map the autophagy pathway. UniProtKB/Swiss-Prot was used to investigate the protein sequences and functional information of the candidate genes. STRING version 10.5 was used to investigate the PPIs of the proteins expressed by the candidate genes. The PPI data were downloaded from the STRING database and uploaded to Cytoscape V3.8.0 for network analysis. The map node size and color of the map were defined in terms of indegree; similarly, the map edge size and color of the map were defined according to the combined score.

## 3. Results

### 3.1. Characterization of the DPTs and DPT-Associated Proteins of Sevoflurane

As described in Table 1, sevoflurane was queried as DB01236 in DrugBank and categorized as an anesthetic agent, a hematologic agent, and a platelet aggregation inhibitor. Sevoflurane is generally applied to induce and maintain general anesthesia, especially in pediatric patients. As shown in Table 2, 23 essential DPTs of sevoflurane were identified using DrugBank.

**Figure 1:**
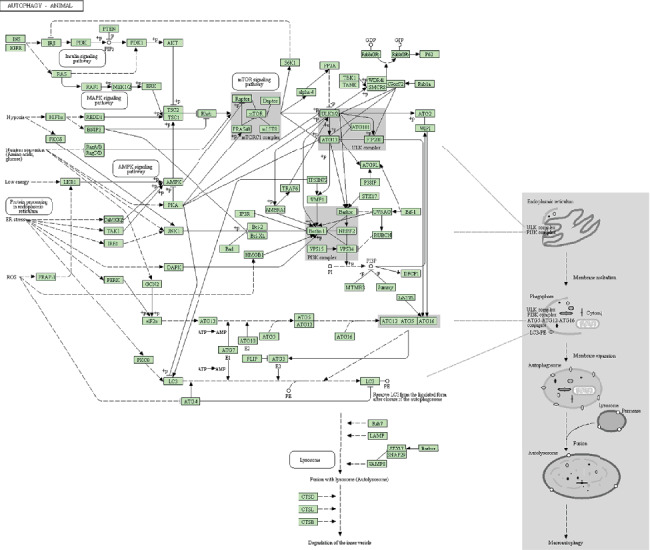


To determine the PPIs of these DPTs in *Homo sapiens*, a single DPT name was used to search STRING version 10.5. After mapping in the STRING database, 77 proteins were identified as DPT-associated genes related to sevoflurane (Figure 1).

### 3.2. Enrichment of Gene Sets Mediated by Sevoflurane

**Figure 2:**
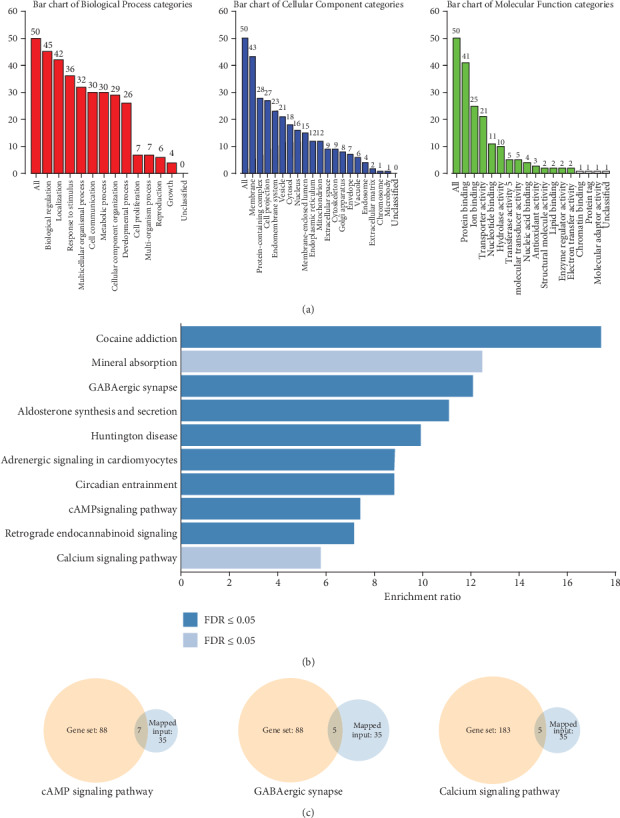
GO enrichment analysis of 77 proteins associated with DPTs by WebGestalt 2019. Only 50 user IDs of 77 proteins associated with DPTs were mapped to the unique Entrez Gene IDs. WebGestalt 2019 was used to evaluate the biological process (red column), cellular component (blue column), and molecular function (green column) categories (a). Among the 50 unique Entrez Gene IDs, 35 were annotated to the selected functional categories and reference list, which were used for the enrichment analysis. The bar chart shows the top 10 pathways in which 35 IDs were enriched (b). The Venn diagram shows that 7 genes in the cAMP signaling pathway, 5 genes in the GABAergic synapse, and 5 genes in the calcium signaling pathway were mapped in the gene set (c).

To investigate the functional properties of the sevoflurane-mediated gene sets, we performed the KEGG pathway enrichment analysis using WebGestalt 2019. The resulting gene list (Figure 1) contained 77 user IDs; of these user IDs, 50 were unambiguously mapped to the unique Entrez Gene IDs, and 27 could not be mapped to any Entrez Gene ID. As shown in Figure 2(a), the Gene Ontology (GO) Slim summary was based on the 50 unique Entrez Gene IDs. In total, 50 genes were included in the biological process, cellular component, and molecular function categories. Among the 50 unique Entrez Gene IDs, 35 IDs were annotated to the selected functional categories and also in the reference list, which were used for the enrichment analysis. The reference list was mapped to 61,506 Entrez Gene IDs, and 7469 IDs were annotated to the selected functional categories that are used as the reference for the enrichment analysis. Benjamini and Hochberg (BH) [19] was used to control the false discovery rate (FDR) of enrichment analysis. As described in Figure 2(b) and Table 3, the top 10 KEGG pathways were as follows: Huntington's disease (17 genes); aldosterone synthesis and secretion (5 genes); retrograde endocannabinoid signaling (5 genes); calcium signaling (5 genes); GABAergic synapse (5 genes); cAMP signaling pathway (7 genes); cocaine addiction (4 genes); adrenergic signaling in cardiomyocytes (6 genes); circadian entrainment (4 genes); and mineral absorption (3 genes). After 35 annotated genes were mapped to a gene set, 7 genes were enriched in the cAMP signaling pathway, 5 genes in GABAergic synapse, and 5 genes in the calcium signaling pathway (Table 3), which are shown in a Venn diagram in Figure 2(c). Three candidate pathways including the GABAergic synapse, cAMP signaling pathways, and calcium signaling pathway were further selected to identify the candidate DPT-associated genes.

### 3.3. Visualization of the Sevoflurane-Linkage Networks by Cytoscape

**Figure 3:**
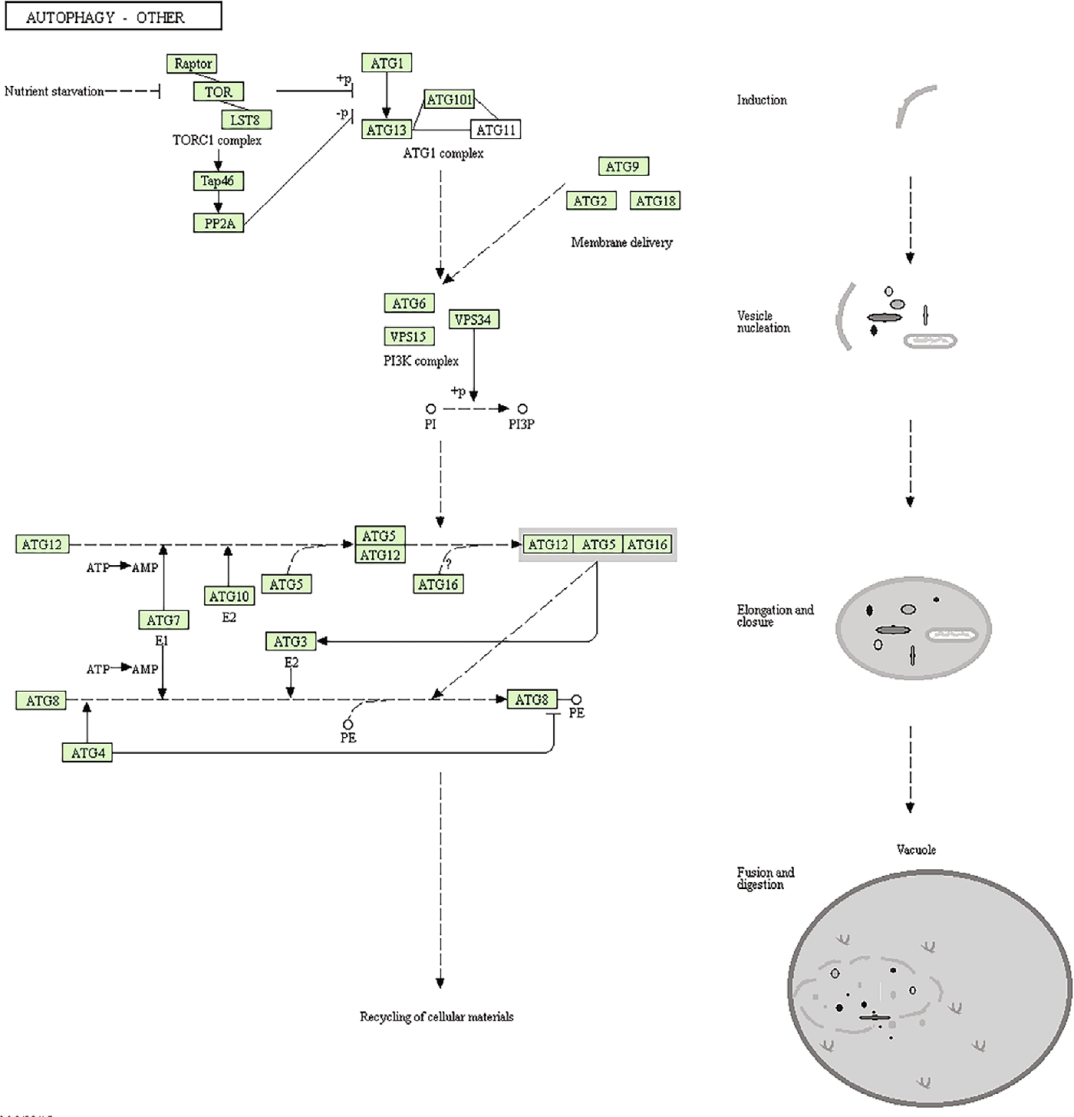


**Figure 4:**
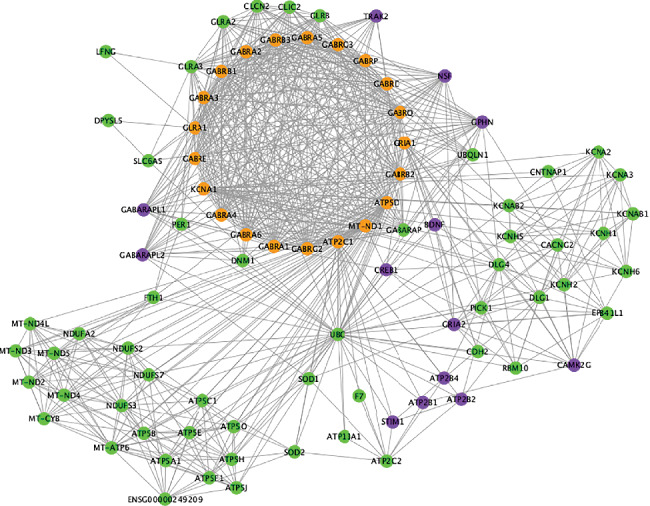
PPI network of 23 DPTs and 77 interacted proteins. The PPI network of 23 DPTs and 77 interacted proteins was generated using the Cytoscape software. Node color was mapped to different kinds of proteins. Green presented DPTs of sevoflurane. Orange meant DPT-associated proteins. Purple showed the proteins enriched in GABAergic synapse, calcium signaling, and cAMP signaling.

**Figure 5:**
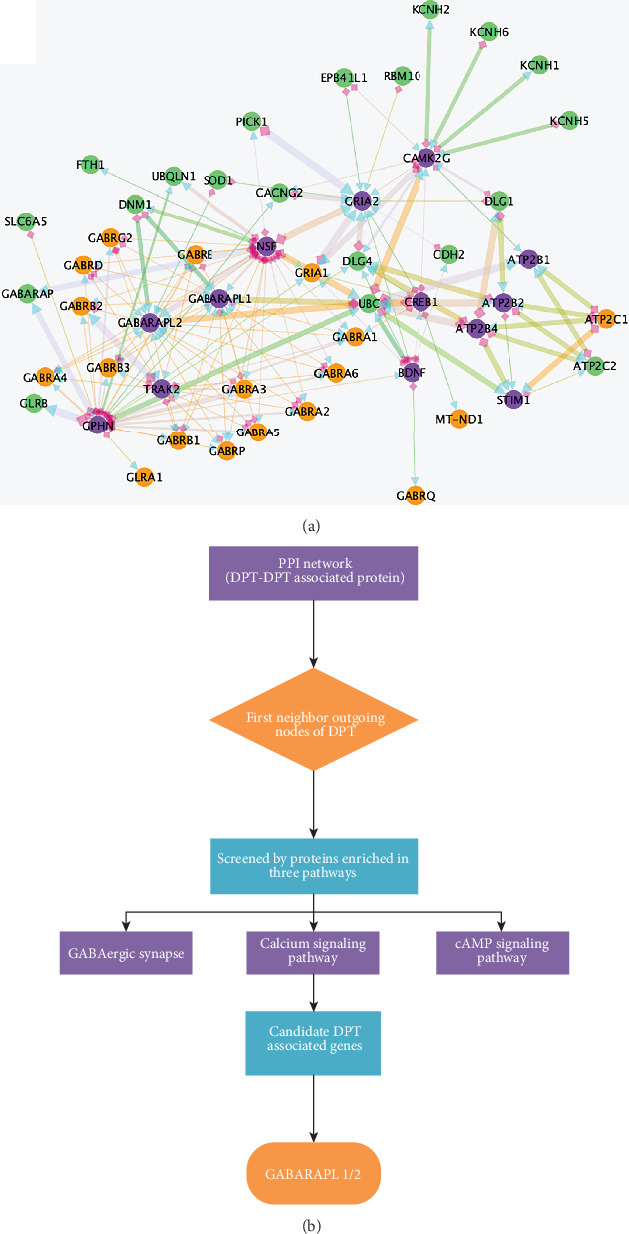
Subnetwork analysis of proteins enriched in candidate signaling. PPI subnetwork was constructed using proteins included in the GABAergic synapse, calcium signaling, and the cAMP signaling pathway with the first neighbor interacted proteins. Node color was mapped to different kinds of proteins. Green presented DPTs of sevoflurane. Orange meant DPT-associated proteins. Purple showed the proteins enriched in GABAergic synapse, calcium signaling, and cAMP signaling. Edge size was mapped to experimentally determined interactions with low values to small size, and edge color was mapped to the combined score with low values to bright colors. Blue arrow indicated target. Pink diamond showed source (a). Flow diagram of screening candidate DPT-associated genes (b).

A relevant biological network containing the DPTs and their associated genes (Figure 3) was constructed using Cytoscape version 3.8.0. The major associated-genes, namely, GABARAP, NSF, DNM1, GABARAPL2, UBQLN1, TRAK2, GABARAPL1, GLRA2, CLCN2, GLRB, GPHN, GLRA3, and CLIC2, were shown to heavily interact with DPTs (Figure 4). As shown in Figure 5(a), 13 genes, namely, NSF (24 PPIs), CAMK2G (9 PPIs), ATP2B1 (3 PPIs), ATP2B2 (10 PPIs), GRIA2 (29 PPIs), BDNF (12 PPIs), ATP2B4 (6 PPIs), GPHN (26 PPIs), GABARAPL1 (20 PPIs), TRAK2 (15 PPIs), CREB1 (8 PPIs), STIM1 (5PPIs), and GABARAPL2 (19 PPIs), were involved in the GABAergic synapse, cAMP signaling pathways, and calcium signaling pathway. These 13 candidate genes were searched in GeneCards one by one. Further analysis revealed that GABARAPL1 and 2 were involved in the autophagy pathway. Autophagy is believed to protect against neurodegeneration [7]. Besides, GABARAPL1 and 2 were identified to be the major targets of sevoflurane-associated DPTs in the subnetwork shown in Figure 5(a). Thus, GABARAPL1 and 2 were selected for further investigation (Figure 5(b)).

### 3.4. Association between GABARAPL1 and 2 and Autophagy-Related Proteins (ATGs)

**Figure 6:**
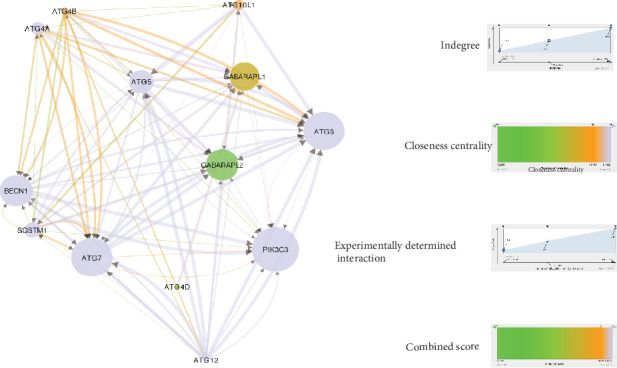
PPI network of GABARAPL1 and 2 with ATG. The PPI network of GABARAPL1 and 2 was built using Cytoscape according to the data from the STRING database. Node size was mapped to indegree with low values to small sizes, and node color was mapped to closeness centrality with low values to bright colors. Edge size was mapped to experimentally determined interactions with low values to small size, and edge color was mapped to combined colors with low values to bright colors. Arrow indicates target.

**Figure 7:**
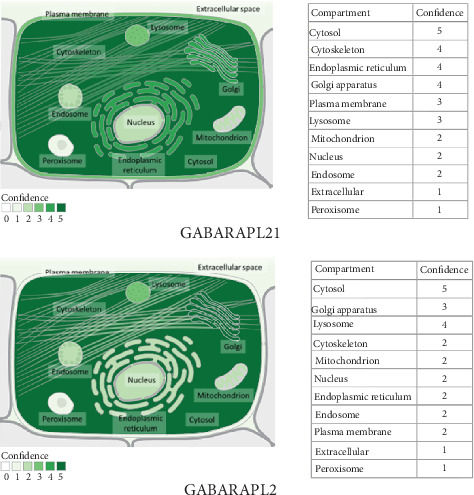


**Figure 8:**
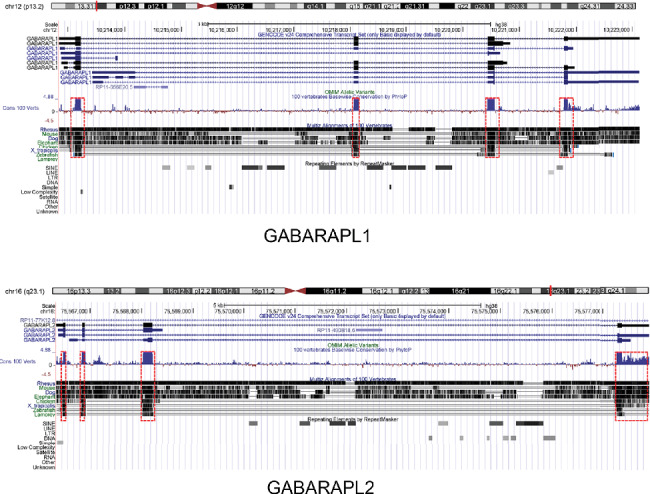


As shown in Figure 6(a), the cascade of ATGs is involved in phagophore formation, elongation, and fusion of lysosomes and autophagosomes. The initiation of autophagy is controlled by the vacuolar protein sorting 34 (VPS34, also known as PIK3C3) complex, which contains beclin-1 (a mammalian ortholog of the yeast ATG6, also called as BECN1) and VPS34. During the phagophore formation, the ATG5-ATG12-ATG16L1 complex requires catalytic reaction under ATG7 and ATG10 to perform catalysis, promoting elongation of autophagosomes. Golgi-associated ATPase enhancer of 16 kD (GATE-16) is cleaved by the AG4A complex with ATG7 and ATG3 to form GATE-16-II, which also supports the elongation of autophagosomes. Microtubule-associated protein 1 light chain 3 (LC3) is first cleaved via ATG4B to form LC3-I and then conjugated by ATG7 and ATG3 to form LC3-II. Lipidated LC3 accumulates in autophagosomal membranes, driving autophagic degradation. The autophagic pathway identified by KEGG pathway enrichment analysis revealed that the ATG8 family is involved in the closure, hemifusion, or transport to promote autophagosome maturation (Figures 7(a) and 7(b)). The network (Figure 8) derived from the STRING database showed that GABARAPL 1 and 2 strongly interact with ATGs including ATG3, ATG4A, ATG4B, ATG4C, ATG4D, ATG5, ATG7, ATG12, BECN1, and PIK3C3 (Figure 6).

### 3.5. GABARAPL1 and 2 Characteristics Identified by Bioinformatics Analysis

**Figure 9:**
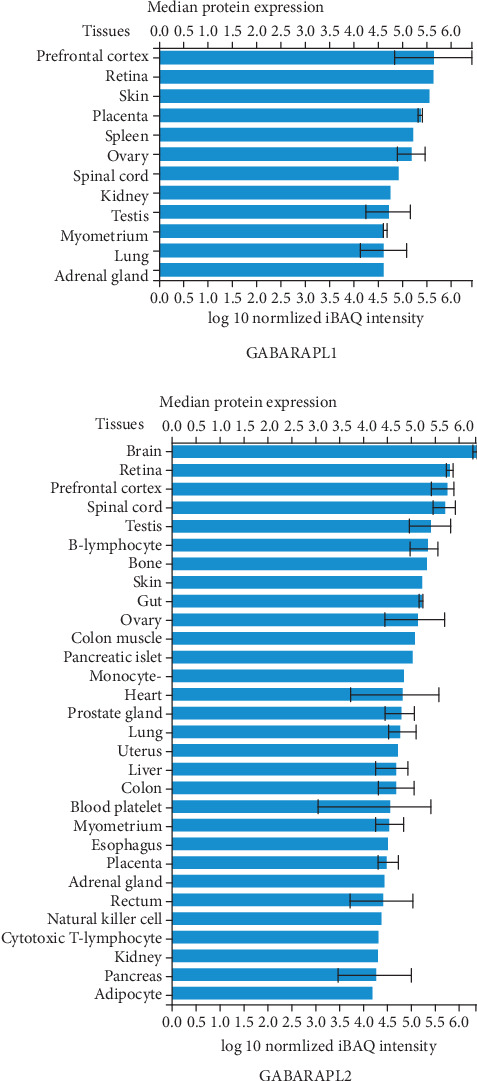


The GABARAPL1 and 2 genes were identified at chr12 (P13.2): 10212805-10223130 and chr16 (q23.1): 75566351-75577881, respectively. Among 100 vertebrates, the 4 exons of GABARAPL1 and 3 exons of the GABARAPL2 were strongly conserved from rhesus monkeys, mice, and dogs to zebrafish (Figure 9(a)). GABARAPL1 and 2 were identified as GC12P010212 and GC16P075566, respectively, using the GeneCards database. The aliases of GABARAPL1 were APG8-LIKE, APG8L, ATG8B, ATB8L, ATG8, GEC-1, and GEC1. Moreover, the aliases of GABARAPL2 were ATG8, ATG8C, GATE-16, GATE16, GEF-2, and GEF2. The GABARAPL1 gene was found to be localized in such subcellular compartments, including the cytosol, cytoskeleton, endoplasmic reticulum, and lysosome. GABARAPL2 was found to be expressed in the cytosol, Golgi apparatus, and lysosomes (Figure 9(b)). GABARAPL1 and 2 mRNAs were found to be expressed in the brain, including areas from the cortex to the hippocampus (Figure 9(c)). GABARAPL1 and 2 proteins were found to be highly expressed in the brain, especially in the frontal cortex (Figure 9(d)).

## 4. Discussion

Using bioinformatics analysis in this study, we found that the dual effect of sevoflurane on neurons is associated with GABARAPL1 and 2. Besides, our results also supported a role for autophagy in regulating sevoflurane-induced neurotoxicity or its neuroprotective effects in different settings, corroborating the results of previous studies [20–22]. Recent reports have suggested that autophagy is implicated in sevoflurane-induced neurotoxicity [12, 20]. In contrast, other data have shown that autophagy plays a crucial role in the neuroprotective effect of preconditioning or postconditioning with sevoflurane on cerebral ischemia [4, 23–25]. With regard to the dual effect of sevoflurane on neurons, our findings further identified the importance of the autophagic network, particularly GABARAPL1 and 2.

Autophagy has been demonstrated to have dual functions [26]. On the one hand, autophagy is beneficial for cell survival. On the other hand, prolonged activation of autophagy beyond a threshold may act as a toxin, resulting in cell death. Autophagy is characterized by the formation of autophagosomes, which is driven by a set of ATGs [8]. In mammalian cells, the ATG8 family includes microtubule-associated protein 1 light chain 3*β* (also known as LC3) and GABARAP, as well as GABARAPL1 and 2 [27]. In this study, GABARAPL1 and 2 were identified to be core targets of direct protein associated with sevoflurane in the subnetwork. Further analysis demonstrated that GABARAPL1 and 2 strongly interacted with other core ATGs, such as ATG7, which agreed with a previous report showing the role of the ATG8 family in the elongation and maturation of autophagosomes [27]. Furthermore, our results have revealed that GABARAPL1 and 2 are evolutionarily conserved among numerous vertebrates, highly expressed in the nervous system, and mainly located in the cytosol, lysosome, and the Golgi apparatus. Collectively, these findings indicated that GABARAPL1 and 2 function as regulators of autophagy in the nervous system.

DTome is an authorized database to identify the DPTs of drugs [17]. Proteins enriched in the top 10 pathways interacted with DPTs of sevoflurane from DTome. Because the GABAergic synapse [28], cAMP signaling pathways [29], and calcium signaling [30] are strongly related to neurotoxicity and neurodevelopment, the genes included in these signaling pathways were selected to investigate DPT-associated genes. Interestingly, bioinformatics analysis of the candidate genes revealed that GABARAPL1 and 2 were core proteins among the DPT-associated proteins. More importantly, GABARAPL1 and 2 not only are known to be homologs of LC3 but also act as crucial regulators of the autophagosomes [31]. A recent study illustrated that proteasome inhibitor treatment caused a dramatic induction of GABARAPL1 rather than GABARAPL2. Knockdown of GABARAPL1 reduced cell survival [32]. In addition, GABARAPL2 was also identified to be involved in Parkinson's disease [33]. Consistent with our analysis, GABARAPL1 and 2 activity was processed by ATG4B, whereas the ATG8 family except GABARAPL1 was inhibited by the inactivity of ATG4B, resulting in neurotoxicity induced by hyperglycemia [34]. However, another new study has shown that GABARAPL1 and 2 played an important role in neurotoxicity with axonal transport deficiency disrupting autophagic flux [35]. In line with our finding, current data supported that GABARAPL1 and 2 may regulate autophagy flux to decide the dual effect of sevoflurane on neurons.

Despite our data originating from authorized databases, including DTome and GeneCards, and STRING, this study has several limitations. Most importantly, the results of the bioinformatics analysis should be further demonstrated using experimental approaches. GABARAPL1 and 2 should be identified by preconditioning and postconditioning brains with sevoflurane in animals suffering from ischemia and by sevoflurane exposure during early or late life. A recent report has suggested that exposing aged rats to sevoflurane leads to the downregulation of LC3, a homolog of GABARAPL1 and 2 [20]. The dual effect of sevoflurane on neurons may be controlled by GABARAPL1 and 2. Thus, it is necessary to perform further experiments to determine if there are DPTs of sevoflurane upstream of GABARAPL1 and 2. This study was designed to investigate only one of the many available molecular mechanisms involved in the dual effect of sevoflurane. Finally, other potential roles of DPT-associated genes enriched in the top 10 pathways other than the GABAergic synapse and cAMP signaling pathways may play a role in regulating the dual effect of sevoflurane on neurons.

Sevoflurane plays a dual role in neuroprotection during brain ischemia and neurotoxicity induced by sevoflurane exposure during early or late life. Our work suggested that sevoflurane controls autophagy flux by targeting DPTs. Emerging evidence has shown that brain ischemia referred to as excitotoxicity is reduced and limited by the activation of autophagy [10]. Using H4 human neuroglia cells, sevoflurane exposure has been shown to induce neurotoxicity and significantly enhanced the level of LC3-II, and these effects are reserved by inhibiting autophagy [21]. In contrast to these reports, the present study constructed a network of DPT-autophagy proteins and expanded our understanding of autophagy regarding the dual effect of sevoflurane on neurons.

## 5. Conclusion

In summary, this study used bioinformatics analysis to demonstrate that the network of sevoflurane-DPT-GABARAPL1 and 2 associated with autophagy is responsible for the dual effect of sevoflurane on neurons.

## Figures and Tables

**Table 1:** Characteristics of sevoflurane using DTome.

DB_ID	Name	Groups	Categories	Indication
DB01236	Sevoflurane	Approved, vet approved	Anesthetics Anesthetics, general Anesthetics, inhalation Central nervous system agents Central nervous system depressants Cytochrome P-450 CYP2A6 substrates Cytochrome P-450 CYP2B6 substrates Cytochrome P-450 CYP2E1 substrates Cytochrome P-450 CYP3A4 substrates Ethers Hematologic agents Hydrocarbons, halogenated Nervous system Platelet aggregation inhibitors QTc-prolonging agents (indeterminate risk and risk modifying)	Used for induction and maintenance of general anesthesia in adult and pediatric patients for inpatient and outpatient surgery

**Table 2:** Identification of direct protein targets of sevoflurane using DTome.

Searched_Drug (1/1)			Target (8)		Action
Number	DB_ID	Name	Target_Symbol	UniProt ID	
1	DB01236	Sevoflurane	GABRA1	P14867	Agonist
2	DB01236	Sevoflurane	GLRA1	P23415	Agonist
3	DB01236	Sevoflurane	GRIA1	P42261	Antagonist
4	DB01236	Sevoflurane	KCNA1	Q09470	Inducer
5	DB01236	Sevoflurane	ATP2C1	P98194	Inhibitor
6	DB01236	Sevoflurane	ATP5D	P30049	Other/unknown
7	DB01236	Sevoflurane	MT-ND1	P03886	Unknown
8	DB01236	Sevoflurane	GABRA1/2/3/4/5/6^∗^	P14867/P47869/P34903/P48169/P31644/Q16445	PAM
8	DB01236	Sevoflurane	GABRB1/2/3, GABRD, GABRE^∗^	P18505/P47870/28472, Q14764, P78334	PAM
8	DB01236	Sevoflurane	GABRG1/2/3, GABRP, GABRQ^∗^	Q8N1C3/P18507/Q99928, O00591, Q9UN88	PAM

GABRA1: gamma-aminobutyric acid receptor subunit alpha-1; glycine receptor subunit alpha-1; glutamate receptor 1; potassium voltage-gate channel subfamily A member 1; calcium-transporting ATPase type 2C member 1; ATP synthase subunit delta, mitochondrial; NADH-ubiquinone oxidoreductase chain 1; ^∗^GABA-A receptor (anion channel); PAM: positive allosteric modulator.

**Table 3:** TOP 10 pathway enrichment from 77 interactive proteins analyzed by WebGestalt.

ID	Pathway	Gene	Expect	FDR
hsa05030	Cocaine addiction	CREB1/DLG4/GRIA2/BDNF	0.22960	4.1*e*-03^∗^
hsa04978	Mineral absorption	CLCN2/FTH1ATP2B1	0.239	5.49*e*-02
hsa04727	GABAergic synapse	GPHN/GABARAPL2/GABARAPL1/NSF/TRAK2	0.41240	4.1*e*-03^∗^
hsa04925	Aldosterone synthesis and secretion	ATP2B1/ATP2B2/ATP2B4/CAMK2G/CREB1	0.44990	4.1*e*-03^∗^
hsa05016	Huntington disease	DLG4/NDUFS7/NDUFA2/NDUFS2/NDUFS3/BDNF/SOD1/SOD2/CREB1	0.90440	1.0*e*-4^∗^
hsa04261	Adrenergic signaling in cardiomyocytes	CACNG2/CREB1/ATP2B1/ATP2B2/ATP2B4/CAMK2G	0.67480	4.1*e*-03^∗^
hsa04713	Circadian entrainment	CREB1/GRIA2/PER1/CAMK2G	0.4499	4.03*e*-02^∗^
hsa04024	cAMP signaling pathway	CREB1/GRIA2/ATP2B1/ATP2B2/ATP2B4/BDNF/CAMK2G	0.93250	4.1*e*-03^∗^
hsa04723	Retrograde endocannabinoid signaling	GRIA2/NDUFS7/NDUFA2/NDUFS2/NDUFS3	0.69350	2.67*e*-02^∗^
hsa04020	Calcium signaling pathway	ATP2B1/ATP2B2/ATP2B4/CAMK2G/STIM1	0.85750	5.4*e*-02

## Data Availability

The data used to support the findings of this study are available from the corresponding author upon request.

## Abbreviations

DPT: Direct protein target
PPI: Protein-protein interaction
GABARAPL: Gamma-aminobutyric acid receptor-associated protein-like
GO: Gene Ontology
FDR: False discovery rate
ATG: Autophagy-related protein
LC3: Light chain 3.
